# The degradation of organic compounds impacts the crystallization of clay minerals and *vice versa*

**DOI:** 10.1038/s41598-019-56756-6

**Published:** 2019-12-27

**Authors:** Pierre Jacquemot, Jean-Christophe Viennet, Sylvain Bernard, Corentin Le Guillou, Baptiste Rigaud, Ludovic Delbes, Thomas Georgelin, Maguy Jaber

**Affiliations:** 1 National Museum of Natural History (MNHN), Sorbonne University, CNRS, Institute of Mineralogy, Material Physics and Cosmochemistry (IMPMC – UMR 7590), F-75005 Paris, France; 2 Sorbonne University, CNRS, Laboratory of Molecular and Structural Archeology (LAMS – UMR 8220), F-75005 Paris, France; 3 Lille University, CNRS UMR 8207, UMET, F-59655 Villeneuve-d’Ascq, France; 4 Sorbonne University, IMPC, F-75005 Paris, France; 5 CNRS, Center of Molecular Biophysics, F-45071 Orléans, France

**Keywords:** Geochemistry, Palaeontology, Mineralogy, Astrobiology

## Abstract

Expanding our capabilities to unambiguously identify ancient traces of life in ancient rocks requires laboratory experiments to better constrain the evolution of biomolecules during advanced fossilization processes. Here, we submitted RNA to hydrothermal conditions in the presence of a gel of Al-smectite stoichiometry at 200 °C for 20 days. NMR and STXM-XANES investigations revealed that the organic fraction of the residues is no longer RNA, nor the quite homogeneous aromatic-rich residue obtained in the absence of clays, but rather consists of particles of various chemical composition including amide-rich compounds. Rather than the pure clays obtained in the absence of RNA, electron microscopy (SEM and TEM) and diffraction (XRD) data showed that the mineralogy of the experimental residues includes amorphous silica and aluminosilicates mixed together with nanoscales phosphates and clay minerals. In addition to the influence of clay minerals on the degradation of organic compounds, these results evidence the influence of the presence of organic compounds on the nature of the mineral assemblage, highlighting the importance of fine-scale mineralogical investigations when discussing the nature/origin of organo-mineral microstructures found in ancient rocks.

## Introduction

The ancient fossil record carries key information on the first steps of the history of life on Earth^1–3^. Yet, although ancient microfossils may still comprise (partially preserved) biogenic organic material^4–10^, the identification of traces of life in ancient rocks has always been fraught with difficulties pertaining to fossilization and burial-induced degradation processes^11–14^. As a result, the interpretation of the Archean palaeobiological record is full of controversies, among which the most famous one is centered on filamentous microstructures found in black chert veins within the 3.46 Ga Apex Basalt (Pilbara Craton, Western Australia)^15,16^. For more than 30 years now, these microstructures have been considered by some authors as the most ancient remains of life on Earth, based on their morphologies and isotopic signatures^17,18^. Yet, besides contamination issues^19,20^, it has recently been argued, based on nanoscale chemical imaging, that these filamentous microstructures are not microfossils (i.e. true body fossils of microorganisms), but rather abiotic stacks of clay minerals fortuitously arranged in roughly filamentous patterns and interleaved with nanoscale mineral phases and organic particles^21–23^.

Laboratory experiments may provide clues regarding the potential impact that hydrothermal/diagenetic processes have had on these microstructures, thereby potentially shedding new light on their origin^14,24^. Although pioneered experimental studies investigated the interactions between biogenic organic compounds and pure silica^25–28^, phosphates^29–31^ or oxides^32,33^, the chemical evolution and mineralogical evolution that may undergo a mixture of reactive biomolecules (i.e. prone to degradation) and clay minerals during advanced fossilization processes remain poorly constrained. In the present study, we experimentally submitted a mixture of RNA (i.e. the most emblematic organic macromolecule of the living world and the central molecule of the ‘RNA world’ hypothesis^34,35^) and a gel of Al-smectite stoichiometry (i.e. a clay mineral ubiquitously distributed in the continental crust^36^) to experimental hydrothermal conditions at 200 °C for 20 days within initially pure water. Additional experiments were conducted in the absence of the gel or in the absence of RNA to serve as controls.

## Results and Discussion

**Figure 1:**
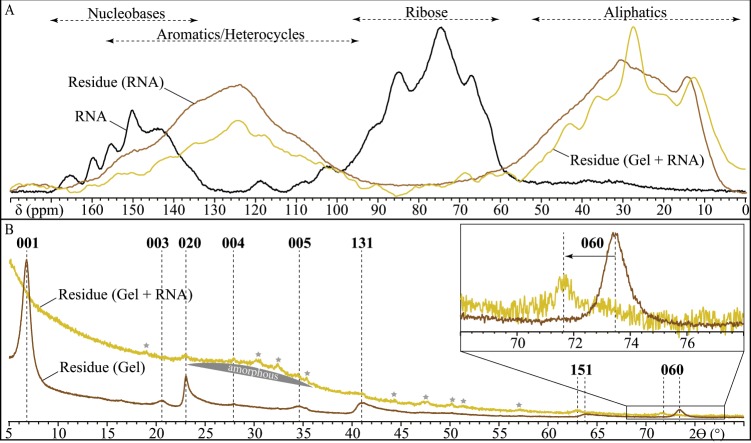
NMR and XRD characterization of the experimental residues. (**A**) CP MAS-^13^C-NMR spectra of the residues of experiments conducted in the presence or absence of a gel of Al-smectite stoichiometry. The CP MAS-^13^C-NMR spectrum of RNA is also shown for comparison. (**B**) Powder XRD patterns of the residues of experiments conducted in the presence or absence of RNA. Values correspond to hkl reflections of clay minerals. Stars correspond to peaks attributed to phosphates.

**Figure 2:**
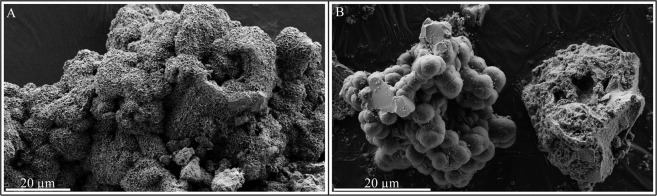
SEM characterization of the experimental residues. (**A**) SEM image of the montmorillonite produced during the experiments conducted in the absence of RNA. (**B**) SEM image of a fraction of the residues of experiments conducted in the presence of RNA, showing a cluster/pile of carbon-free micrometric spheres of pure amorphous silica (left) and a pluri-decimicrometric heterogeneous aggregate (right).

Results evidence that RNA underwent significant transformations during the experiments (Fig. 1A). While the CP MAS-NMR signature of RNA is dominated by the signals of ribose (signal between 60 and 105 ppm) and nucleobases (signal between 140 and 170 ppm), the CP MAS-NMR signatures of the residues of experiments conducted either in the presence or absence of a gel of Al-smectite stoichiometry are dominated by aromatic/heterocyclic and aliphatic carbons (signals between 100 and 150 ppm and between 55 and 70 ppm, respectively). In absence of RNA, the gel of Al-smectite stoichiometry evolved into a crystallized montmorillonite^36^ (Figs. 1B and 2A). In contrast, only a small amount of crystalline phyllosilicates is detected together with mainly amorphous phases in residues of experiments conducted in the presence of RNA (Fig. 1B). Surprisingly, the phyllosilicate that crystallizes is not montmorillonite: instead of exhibiting the 060 reflection of dioctahedral montmorillonite at 1.498 Å, this phase exhibits a 060 reflection at 1.532 Å, i.e. a value more typical of 2:1 trioctahedral Mg-rich smectites^36^ (Fig. 1B).

**Figure 3:**
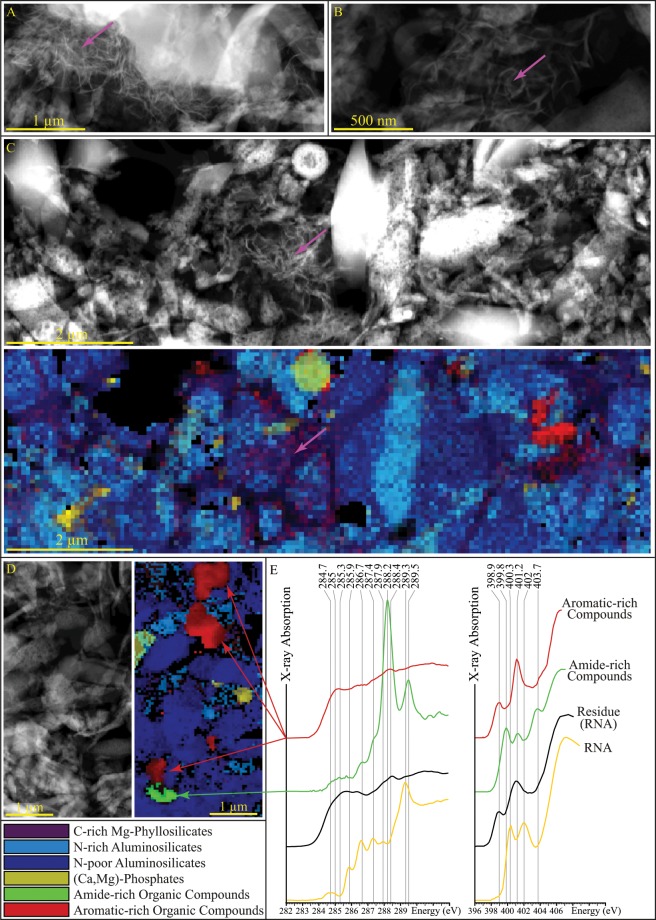
STEM and STXM characterization of the experimental residues. (**A**–**D**) STEM-HAADF images of fractions of the residues of experiments conducted in the presence of RNA and corresponding maps (**C**,**D**). Clay minerals more or less associated with organic carbon are clearly visible (pink arrows). (**E**) XANES spectra at the C and N K-edges of organic compounds encountered in the residues. At the C K-edge, energy values (in eV) correspond to typical absorptions of quinones or conjugated cyclic amides (284.7), alkenes and aromatics (285.0), conjugated cycles (285.3), heterocycles (285.9), imines and nitriles (286.7), phenols and ketones (286.7/287.4), aliphatics (287.9), amides (288.2), carboxylics (288.4) and hydroxyls (289.3/289.5)^42,44^. Due to overlapping energies of several resonances, absorption peaks at the N K-edge cannot be univocally assigned to given functional groups, but absorption features below 400 eV generally indicate the presence of imine, nitrile and pyridinic N while absorption features above 400 eV generally indicate the presence of amide, nitro and pyrrolic N^44,45^. The spectra of RNA and of the residue of the experiments conducted in the absence of the gel of Al-smectite stoichiometry are shown for comparison.

SEM investigations reveal that, rather than pure clay minerals (Fig. 2A), the presence of RNA leads to complex organo-mineral residues composed of (i) clusters of carbon-free micrometric spheres of pure amorphous silica, and (ii) heterogeneous aluminosilicate-rich aggregates containing Al, Mg, P, C and N in addition to Si (Fig. 2B). The heterogeneous nature of these aggregates is highlighted at the sub-micrometer scale by TEM imaging and EDX mapping of cryo-ultramicrotome sections (Fig. 3A–D): these aggregates are composed of submicrometric particles of more or less N-rich amorphous aluminosilicates and (Ca,Mg)-phosphates associated with relatively rare Mg-smectites that appear to be in close association with organic carbon (Fig. 3C). This organic material might be either adsorbed onto or bonded to surfaces (lateral or basal) of these Mg-smectites or trapped within their interlayer spaces^37^.

Organic carbon also occurs within these residues as submicrometric particles of various chemical compositions as indicated by STXM-based XANES spectroscopy (Fig. 3E): (i) aromatic-rich organic particles (N/C ~ 0.15) containing a significant proportion of conjugated cycles and heterocycles (peaks at 285, 285.4 & 285.9 eV, respectively) and traces of carboxylics (peak at 288.4 eV), and (ii) N-rich organic particles (N/C ~ 1) dominated by amide and hydroxyl groups (absorption peaks at 288.2 & 289.5 eV, respectively). These N-rich organic particles are absent from the residues of experiments conducted in the absence of the gel of Al-smectite stoichiometry. In fact, these very homogeneous residues (N/C ~ 0.15) exhibit a XANES spectrum very similar to that of the heterocyclic, (poly)aromatic organic particles.

Although the information related to the original chemical structure of RNA was lost during the experiments, amide-rich organic compounds were produced in experiments conducted in the presence of a gel of Al-smectite stoichiometry. Similar compounds have been reported within ancient microfossils and interpreted as products of the degradation of peptidic compounds^6,8^. This is not the case here as RNA does not contain peptidic moieties but rather conjugated cyclic amides. Such preservation of amide moieties occurred only in the presence of the gel of Al-smectite stoichiometry, suggesting that the presence of this initially amorphous inorganic phase limited the molecular degradation of RNA, consistently with results of experiments conducted with silica^27^.

In addition to the influence of a mineral phase on the degradation of organic compounds, the present results evidence the influence of the presence of organic compounds on the final mineral assemblage. Instead of pure clay minerals as in the absence of RNA, the residues of experiments conducted in the presence of RNA comprise amorphous silica, aluminosilicates and nanophosphates associated with rare nanoscale clay minerals. As illustrated here, although in some cases the presence and the nature of organic materials composing ancient putative microfossils may not be informative enough to discuss their possible biogenicity, the nature of the organo-mineral assemblage may be quite rich in information. Although additional experiments would be required to properly constrain the origin of the Apex microstructures, the present study highlights the importance fine-scale mineralogical investigations, as suggested earlier^21–23^, when discussing the nature/origin of organo-mineral microstructures found in ancient rocks.

## Materials and Methods

### Starting materials

Pure powders of yeast RNA (SIGMA-ALDRICH) and a hydrogel of Al-smectite stoichiometry synthetized in the lab were used for the present experiments. The hydrogel was obtained by adding 25.1 mL of a solution of aluminium nitrate (81.2 g/L) and 4.01 mL of a solution of magnesium nitrate (100.45 g/L) into a 15 mL solution of ethanol containing 0.1 mol of TEOS (tetraethylorthosilicate) and 0.005 mol of CaCO_3_. An ammoniacal solution (33%) was then added until gelification. The gel was stored 1 night at room temperature and heated 1 day at 200 °C and 1 day at 650 °C.

### Fossilization experiments

Experiments were conducted in 25 mL PARR Reactors under hydrothermal conditions using 50 mg of RNA, 100 mg of the gel of Al-smectite stoichiometry and 5 mL of water. The Ti reactor were placed at 200 °C for 20 days before being cooled at room temperature. Residues were dried overnight at 60 °C. Additional experiments were also conducted with either no mineral or no RNA to serve as controls. For TEM and STXM measurements, residues were washed 3 times with water and dichloromethane/methanol (1:1) to remove the soluble organic fraction. All experiments were triplicated to ensure reproducibility.

### X-Ray diffraction

X-Ray diffraction patterns were acquired using a PANALYTICAL X’PERT PRO diffractometer (IMPMC, Paris) operating at 40 kV and 40 mA with a Co anode (Kα at 1.79 Å). Analyses were performed on finely ground powders deposited on a silicium sample holder. The angular range in 2θ was 5–80° with a step size of 0.017° for a total counting time per sample of 2 hours.

### Scanning electron microscopy

SEM investigations were performed on powders deposited on carbon tape using SEM-FEG ZEISS ULTRA 55 (IMPMC, Paris).

#### CP MAS-^13^C-NMR

Cross polarization (CP) ^13^C nuclear magnetic resonance (^13^C-NMR) experiments were performed with a magic-angle spinning (MAS) probe 1 H/X at 14000 kHz in a BRUCKER AVANCE 3 500 MHz (IMPC, Paris) operating at 125.77 MHz. Samples were sealed in a 4.0 mm zircon rotor. Chemical shifts were calibrated using the carboxyl signal of adamantane (38.52 ppm). The ^13^C Cross-Polarization spectra were acquired with a ramp-CP contact time of 1 ms and a 1.5 s of recycle delay.

#### Sample preparation for TEM and STXM

Cryo-ultramicrotome sections were prepared for STXM and TEM characterization using the LEICA ultramicrotome available at UMET (Lille, France). Experimental residues were mixed with 0.1 ml of water-ethanol (50/50%_Vol_) before being frozen in liquid nitrogen at −160 °C. After cutting, the ultrathin slices of residues were deposited on holey carbon film TEM grids before being exposed to ambient temperature.

#### Transmission electron microscopy

Scanning transmission electron microscopy (STEM) and EDS mapping were performed using a THERMOFISHER TITAN THEMIS 300 microscope operated at 300 kV (CCM – Lille). Hyperspectral EDS data were obtained using the super-X detector system equipped with four windowless silicon drift detectors. These detectors have a high sensitivity for light elements and allow a high counting rate of the carbon, nitrogen and oxygen X-rays. The probe current was set at 600 pA with a dwell time at 10 µs per pixel. A key aspect of this work is the post-processing of the hyperspectral data, performed using the HYPERSPY python-based package^38^. The signal was first denoised using principal component analysis. Then, the EDS spectra at each pixel were fitted by a series of Gaussian functions and a physical model for background/bremsstrahlung. The integrated intensities of the Gaussian functions were used to quantify the compositions thanks to the Cliff-Lorimer method, using experimentally determined k-factors. Absorption correction was taken into account using an experimental thickness map obtained by integration of plasmon absorption bands in EELS. This step was mandatory to correct for the re-absorption within the sample of the carbon, nitrogen and oxygen X-rays. Finally, end-member phases (smectites, phosphates, amorphous silica, organic compounds) were identified and their spectra used as inputs for linear combination fitting (multiple linear least square fits). Pixels of similar composition were given the same colors scaled as a function of the proportion of each phase.

#### Scanning transmission X-ray microscopy

STXM/XANES investigations were conducted using the HERMES STXM beamline^39,40^ at the synchrotron SOLEIL (Gif-sur-Yvette, France). Carbon contamination on beamline optics is constantly removed thanks to a continuous flow of pure O_2_. The well-resolved 3p Rydberg peak of gaseous CO_2_ at 294.96 eV and the 1 s → π* photoabsorption resonance of gaseous N_2_ at 400.89 eV were used for energy calibration. Collecting image stacks at energy increments of 0.1 eV with a dwell time of ≤ 1 ms per pixel prevented irradiation damage^41^. The C- and N-XANES spectra shown here were normalized to the carbon and nitrogen quantities using QUANTORXS^42^. N/C values were estimated at +/− 0.02^43^ using QUANTORXS^42^.
